# Scalable Microwires through Thermal Drawing of Co-Extruded Liquid Metal and Thermoplastic Elastomer

**DOI:** 10.3390/ma17112770

**Published:** 2024-06-06

**Authors:** Pranjal Khakse, Falco Dangers, Rawan Elsersawy, Mohammad Abu Hasan Khondoker

**Affiliations:** Industrial Systems Engineering, Faculty of Engineering and Applied Science, University of Regina, Regina, SK S4S 0A2, Canada; pjkhakse@gmail.com (P.K.); falcodangers@gmail.com (F.D.); rre285@uregina.ca (R.E.)

**Keywords:** liquid metals, co-extrusion, thermal drawing, microwires, stretchable electronics, EGaIn

## Abstract

This article demonstrates scalable production of liquid metal (LM)-based microwires through the thermal drawing of extrudates. These extrudates were first co-extruded using a eutectic alloy of gallium and indium (EGaIn) as a core element and a thermoplastic elastomer, styrene–ethylene/butylene–styrene (SEBS), as a shell material. By varying the feed speed of the co-extruded materials and the drawing speed of the extrudate, it was possible to control the dimensions of the microwires, such as core diameter and shell thickness. How the extrusion temperature affects the dimensions of the microwire was also analyzed. The smallest microwire (core diameter: 52 ± 14 μm and shell thickness: 46 ± 10 μm) was produced from a drawing speed of 300.1 mm s^−1^ (the maximum attainable speed of the apparatus used), SEBS extrusion speed of 1.50 mm^3^ s^−1^, and LM injection rate of 5 × 105 μL s^−1^ at 190 °C extrusion temperature. The same extrusion condition without thermal drawing generated significantly large extrudates with a core diameter of 278 ± 26 μm and shell thickness of 430 ± 51 μm. The electrical properties of the microwires were also characterized under different degrees of stretching and wire kinking deformation which proved that these LM-based microwires change electrical resistance as they are deformed and fully self-heal once the load is removed. Finally, the sewability of these microwires was qualitatively tested by using a manual sewing machine to pattern microwires on a traditional cotton fabric.

## 1. Introduction

Flexible and stretchable electronics are revolutionizing the field of wearable electronics [1]. They offer unique advantages such as conformability to the human body [2]. Historically, mercury was used in some flexible and stretchable electronic applications [3,4], but due to its toxicity [5,6] it is no longer used by researchers in the field of wearable electronics. Recently, liquid metal alloys such as EGaIn (the eutectic alloy of gallium and indium) [7] and Galinstan (a gallium–indium–tin alloy) [8] have been successfully used in flexible and stretchable electronics applications. These gallium-based non-toxic liquid metal (LM) alloys remain in a liquid state at room temperature and offer excellent properties such as high electrical conductivity and mechanical stretchability which make them a perfect candidate for wearable electronics applications. One of the challenges in the field of flexible and stretchable electronics is the fabrication of microwires that can be patterned into 3D structures to manufacture functional devices like soft-matter sensors, reconfigurable actuators, etc. [9]. These LM alloys instantly form an oxide skin with substantially high surface tension when exposed to atmospheric air [10]. Although this oxide skin helps to maintain bare structures such as directly written free-standing micro-components [11], the oxide skin also makes pressure-driven extrusion printing of LM alloys very challenging by forming globes rather than continuous streams [12]. Due to the instantly formed oxide skin, patterning LM structures while encapsulated in an insulated shell is particularly desired [9]. While injecting LM into a hollow prefabricated insulation shell to make LM wires for functional devices has been successfully demonstrated [13,14], this method does not allow the fabrication of microwires due to the extremely high-pressure requirements [5]. Moreover, this two-step fabrication process is not scalable for producing long, high-quality LM microwire with full control of core–shell dimensions. To overcome this problem, researchers have demonstrated 3D printing of core–shell wire systems with LM alloys as core conducting materials with a flexible and stretchable polymer as the insulating shell material. For instance, Khondoker et al. used a thermoplastic elastomer, styrene–ethylene/butylene–styrene (SEBS), as a shell material with EGaIn which can withstand stretching up to 400% without any loss in mechanical or electrical properties [7]. For this, they utilized an in-house feeding system to directly input raw pellets of SEBS [15] and a custom extruder head for the co-extrusion process. Macro-scale LM wires typically can be stretched up to a maximum of 300% without needing excessively large force, as demonstrated in previous studies [16]. On the other hand, LM microwires are expected to be stretched extremely and easily. Moreover, microwires with smaller diameters also offer sewability, which is crucial for wearable textile applications. These attributes underscore microwires as highly promising candidates for incorporation into wearable devices, representing a significant potential for a wide range of enduring applications focused on durability, adaptability, and reliability. Long-term wearable health monitoring systems stand out as one such application area, with microwires seamlessly integrating into devices designed for continuous tracking of vital signs such as heart rate, blood pressure, and temperature, ensuring prolonged wear on the body without discomfort. Additionally, they prove valuable in environmental sensing under harsh conditions, facilitating the monitoring of factors like temperature, humidity, and chemical pollutants in remote or industrial settings. Furthermore, their integration into structural materials enables structural health monitoring in infrastructure, allowing for the detection of changes in strain, stress, or temperature for proactive maintenance and safety enhancements. In underwater exploration, these microwires serve as flexible sensors for long-term monitoring of water conditions, while also contributing to energy harvesting devices for sustainable power generation in remote locations without the need for maintenance. Moreover, they enhance outdoor textiles with features like heating and lighting and find applications in the aerospace and automotive industries for structural health monitoring, collectively expanding the scope of healthcare, environmental monitoring, infrastructure, energy harvesting, and textiles with their soft, stretchable, and flexible attributes. On the other hand, He et al., used a slow-curing sealant, silicone paste, as a shell material with Galinstan to construct 3D circuit networks [16]. While these works successfully demonstrated the use of 3D printing technologies for extremely flexible and stretchable electronics based on LM alloys, the coarser printing resolution of these extrusion-based technologies prevents the use of their printed devices in niche applications where micro-scale wires or channels and small form factors are crucial [17,18]. The constraints of current methods encompass scalability issues, as they do not always facilitate unlimited length production, and the resulting wires may not always qualify as microwires. Therefore, there is a strong need for a scalable process to manufacture LM alloy-based microwires. This article presents a co-axial extrusion process of an LM alloy and a thermoplastic elastomer to fabricate microwires via the subsequent thermal drawing step. This process enables the production of high-quality, flexible, and stretchable LM microwires that can be used in a wide range of wearable electronics applications.

## 2. Experimental Setup

### 2.1. Materials

In this work, as a thermoplastic elastomer, pellets (<5 mm size) of styrene–ethylene/butylene–styrene (SEBS), commercial name: Kraton G1657, were provided by Kraton Polymers U.S. LLC, Houston, TX, USA. Gallium metal (99.99% pure) in the tube and indium metal shots were sourced from Amazon.com (Store: Gallant Metals). Gallium metal cut into small flakes was mixed with indium metal shots at a weight ratio of gallium:indium = 75.5%:24.5% and heated in a glass vial using a hot plate for approximately 30 min. Thus, EGaIn was prepared in-house. For external wiring, enameled copper microwires sourced from Amazon.com (store: Benecreat) were used after removing the insulation coating from the ends.

### 2.2. Methodology

#### 2.2.1. Desktop Pellet Extruder

To extrude SEBS pellets, a single screw desktop filament extruder (Wellzoom, Shenzhen, China), was used. This is a single heating zone extruder that uses a band heater installed on the barrel to melt the polymer. The existing extruder nozzle of the Wellzoom extruder was replaced with a custom nozzle, described below. This inexpensive extruder does not have a properly calibrated control to adjust the extrusion speed. Therefore, before the experiments, a preliminary test was performed to calibrate the extruder knob to precisely control extrusion speed at two different extrusion temperatures (190 °C and 210 °C). For the extrusion of SEBS, the calibration was conducted based on the volumetric unit of mm^3^ s^−1^.

#### 2.2.2. Extruder Nozzle Design

**Figure 1:**
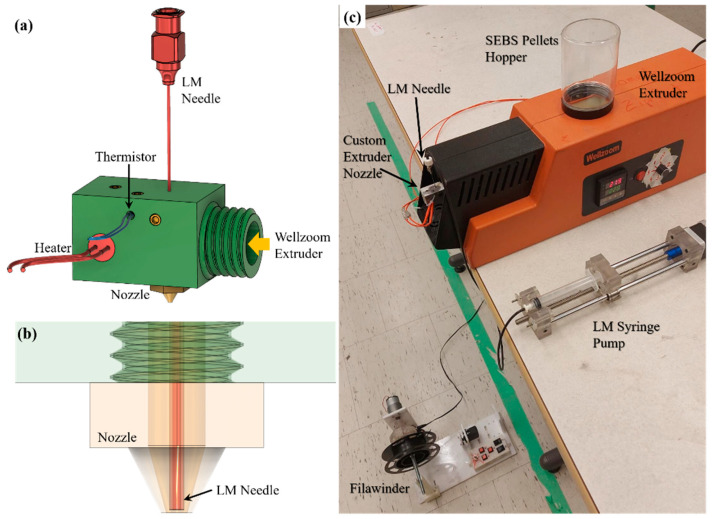
(**a**) 3D model of the custom extruder nozzle depicting the LM needle and nozzle assembly; (**b**) see-through model showing the co-axial needle/nozzle orientation; (**c**) entire setup showing Wellzoom and LM syringe pump on the table with winding system kept on the floor.

As shown in Figure 1a, a co-axial extruder nozzle was designed which was machined by Proto Labs, Inc., Maple Plain, MN. A detailed engineering drawing file of this extruder nozzle is provided in the Section S1 of Supplementary Information (Figures S1 and S2). A 22-gauge, 2″ long reusable stainless-steel needle (Product No. 6710A29) was sourced from McMaster-Carr, Elmhurst, IL to inject the LM alloy.

A 24 V cartridge heater and a 100 kΩ thermistor installed on the custom nozzle were connected to an external temperature controller to maintain the temperature of the nozzle the same as the Wellzoom extrusion temperature. It was ensured that the needle was positioned at the center of the nozzle (as shown in Figure 1b) and stayed immediately inside the nozzle orifice. The size of the nozzle orifice was 1 mm.

#### 2.2.3. Custom Syringe Pump

Inspired by the open-source design of the syringe pump [19], we developed a syringe pump by 3D printing some components and purchasing parts like guide rods, ball bearings, linear bearings, and so on. Then, we used this syringe pump with a 20 mL syringe loaded with LM alloy. The Luer-Lock adapters of the LM syringe were connected to the co-axial needle of the extruder nozzle. Then, another preliminary test was performed to calibrate the stepper motor steps to control the flow of LM alloy through the needle. The flow rate of the LM alloy was calibrated in the unit of μL s^−1^.

#### 2.2.4. Winding System

**Figure 2:**
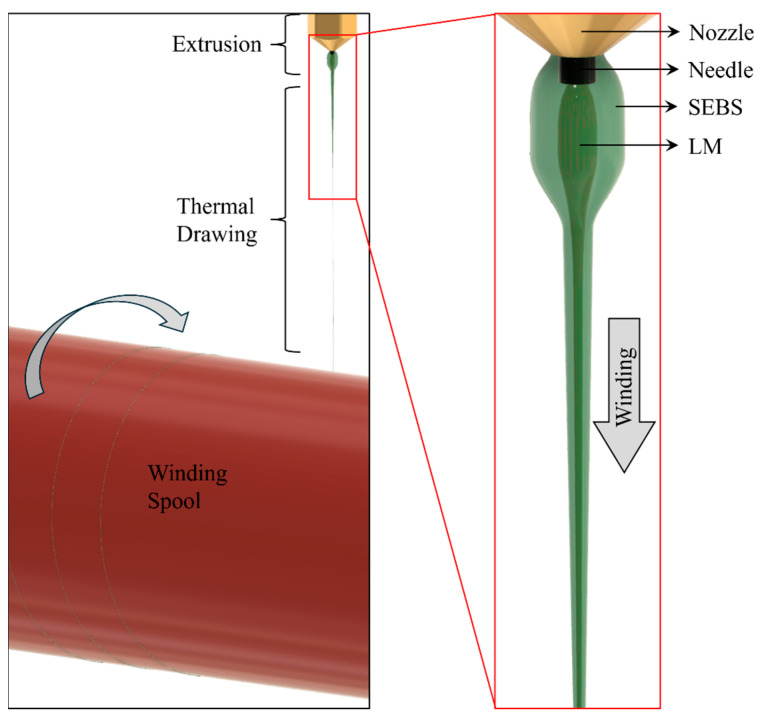
3D model of the system showing the three stages of the manufacturing process starting with extrusion, then thermal drawing, and finally winding spool.

As a winding system, FILAWINDER [20], commercialized by Filastruder, Snellville, GA, USA, was used. The winding speed of this winding system was limited, which was increased by changing the existing gearbox. To further increase the winding speed, a spool of larger diameter (85 mm) was used to wind the drawn microwires. Where the maximum rpm of the existing winding system was around 38 rpm, the maximum rpm was measured to be around 80 rpm after modification. However, this high rpm speed introduces some vibration which was found to affect the quality of the drawn microwire. Therefore, the maximum rpm that was tested in this custom winding system was 67 rpm, which is equivalent to a drawing speed of 300 mm s^−1^ of the microwire. We also tested with 15 rpm, equivalent to 70 mm s^−1^ of drawing speed. In Supplementary Information (Section S2), the tested drawing speeds are further explained. Figure 1c shows the entire experimental setup depicting all the devices used in the production of LM microwires. The winding system was carefully placed very close to the table on which the Wellzoom extruder was located to ensure only a vertically downward drawing force was applied to the extrudates, as shown in Figure 2.

#### 2.2.5. Sewing Machine

A manual SINGER^®^ (Model: STARTTM 1304) sewing machine, bought from Canadian Tire, Regina, SK, Canada, was used for sewing the microwires on fabrics to generate LM patterns.

## 3. Results and Discussion

### 3.1. Core–Shell Dimension

**Figure 3:**
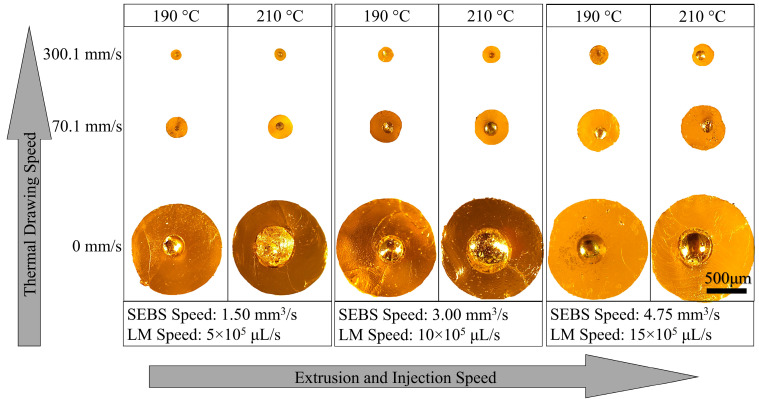
Microscopic images of the cross-sections of the representative LM microwires produced with different extrusion conditions and thermal drawing speeds. LM core with SEBS shell can be seen for all microwires.

By varying the SEBS extrusion speed, LM injection rate, and extrudate drawing speed at different temperatures (210 °C and 190 °C), it was possible to produce microwires with different LM core diameters and SEBS shell thicknesses. The thermal drawing was applied immediately after the materials were extruded to ensure the SEBS melt temperature was still above its upper glass transition temperature (Tg). As shown in Figure S3 of Supplementary Information, while the needle’s internal opening area (A) was occupied by the LM, the nozzle opening area external to the needle (C) was completely filled by the SEBS during extrusion. Immediately after the extrusion, the gap between area A and area C, which formed due to the needle thickness, acted as a buffer zone to accommodate not only the die swell of SEBS but also the slight thickening of LM due to higher surface tension. However, the combined effect of die swell and surface tension was considerably high, such that when the thermal drawing was not applied, the extrudate diameter eventually became much larger than the nozzle diameter. As soon as the thermal drawing was applied, the extrudate dimension substantially decreased, as shown in Figure 3. As discussed in Section S3 of Supplementary Information, the SEBS extrusion speed and LM injection rate were adjusted as per the ratio of area C and area A of Figure S1, which is 3:1. Hence tested extrusion speeds of SEBS were 1.50 mm^3^ s^−1^, 3.00 mm^3^ s^−1^, and 4.75 mm^3^ s^−1^, whereas the LM injections rates were 5 × 105 μL s^−1^, 10 × 105 μL s^−1^, and 15 × 105 μL s^−1^, as calculated in Table S1. By changing the rpm of the winding system, the three tested drawing linear speeds were 0 mm s^−1^, 70.1 mm s^−1^, and 300.1 mm s^−1^.

As shown in Figure 3, the LM/SEBS extrudates produced without any thermal drawing clearly show the LM core with a circular SEBE shell. While the LM cores were found to have some degrees of eccentricity with respect to the SEBS shell, the extrudates did not show any rupture of the plastic shell for potential LM leakage. The extrudates produced at 190 °C extrusion temperature seem to have smaller LM cores when compared to their counterparts produced at 210 °C. This is believed to be caused by the higher viscous drag exerted by the SEBS melt at lower extrusion temperature that dominates the surface tension force of the LM core which tends to form globes rather than a continuous stream [7]. It is also noted that the core/shell dimensions are likely to increase as the extrusion/injection rate increases, although the same nozzle (1 mm) was used. This phenomenon is because of the higher degree of die swell associated with higher extrusion rates of SEBS melt [15,21]. As the thermal drawing (70.1 mm s^−1^) was applied to these extrudates, the circular shape of the core–shell system was maintained, while the core–shell dimensions substantially reduced to around only 20~47% (depending on extrusion speed), as compared to the dimensions of as-extruded wires. However, with a higher drawing speed of 300.1 mm s^−1^, the core–shell dimensions were further reduced to around 10~21% when compared to the as-extruded wires. These size reduction percentages at considerably higher drawing speeds were not as significant as size reduction at lower drawing speeds. This suggests that with higher drawing speed, the materials eventually reach the saturation limit dictated by the surface tension of the oxide skin. Because of this, drawing speeds higher than 300.1 mm s^−1^ resulted in un-uniform core dimension, resulting in occasional breakage of the LM core or rupturing of the SEBS shell during drawing.

**Figure 4:**
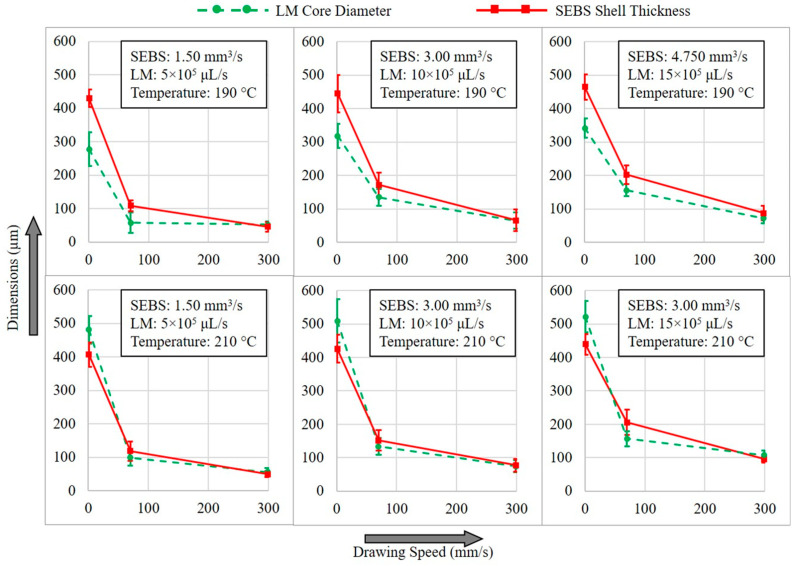
Variation in LM core diameter and SEBS shell thickness of the drawn microwires, depicting the effect of process parameters on the core–shell dimensions.

Figure 4 shows how the core diameter and shell thickness of LM microwires varies with different drawing speeds and extrusion/injection rates at different temperatures. Measurements were taken on the cross-sections of multiple samples produced from the same process parameters. SEBE shell thicknesses were also measured on the cross-section of the same sample at different locations to capture the eccentricity of the LM core with respect to the shell. The smaller error bars of shell thicknesses in Figure 4 exhibit the LM core being located closer to the center of the SEBS shell. As shown in this Figure, it was found that microwires with the most centrally positioned LM core were produced at a lower temperature (190 °C) and higher extrusion and drawing (300.1 mm s^−1^) speeds. Another important finding is that the LM core diameters of as-extruded wires produced at a higher temperature (210 °C) are slightly larger than the shell thicknesses which is because of the lower viscous drag applied by the SEBE melt of higher temperature. However, after thermal drawing, the LM core diameter reduces to smaller than SEBS shell thickness, as seen for microwires produced at a lower temperature (190 °C). In all cases, the core diameter and shell thickness of microwires produced at 300.1 mm s^−1^ drawing speed was very similar, exhibiting narrow size distributions.

### 3.2. Resistivity of Microwire

**Figure 5:**
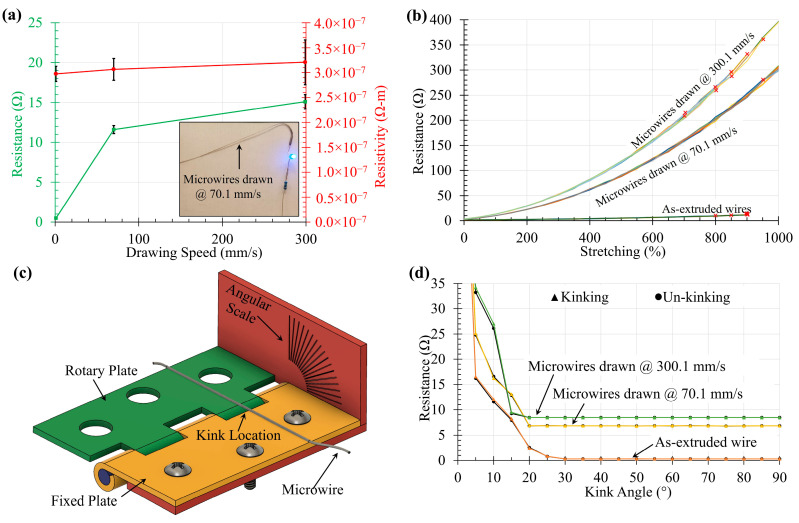
(**a**) Electrical resistance and resistivity of 10 cm long microwires produced with different drawing speeds; conductivity of a microwire drawn at 70.1 mm s^−1^ demonstrated in the inset; (**b**) effect of different degrees of stretching of 2 cm long microwires on their electrical resistance; (**c**) 3D model of custom setup for wire kinking test; (**d**) change in resistance of 6 cm long microwires when subjected to a single kink shown for both kinking and un-kinking process from 90° (perpendicular bending) to 0° (full severed).

To characterize the electrical properties (resistance and resistivity) of the microwires, 10 cm long microwires were first cut and connected with copper wires as end connections. An Arduino-based setup was used to measure the resistance of the microwires using the voltage divider rule. For this purpose, microwires drawn at different speeds but produced with the same SEBS speed of 1.5 mm^3^ s^−1^ and LM rate of 5 × 105 µL s^−1^ were tested. As shown in Figure 5a, the resistance of the microwires was of higher order when drawn at higher speeds. Each data point represents five test specimens. Once the resistance was measured, the resistivity was calculated using the cross-sectional dimensions measured from microscopic images. As expected, although microwires drawn at higher speeds exhibited significantly higher resistance, the estimated resistivity of all wires was found to be in the same order. However, it was noted that the standard deviation of both resistance and resistivity of microwires with smaller core diameters showed substantially wide variations. This can be attributed to the effect of slowly formed oxide skin inside the SEBS channel due to air diffusion. The electrically less conductive, randomly formed oxide skin causes occasional interruption of the LM core, resulting in variation in electrical properties [22].

### 3.3. Electrical Property with Stretching

To study the effect of stretching (up to 1000%) on the electrical properties of the microwires, 2 cm long microwires produced with the same SEBS speed of 1.5 mm^3^ s^−1^ and LM rate of 5 × 105 µL s^−1^ were tested. Ten (10) microwires drawn at different speeds were subjected to incremental stretching performed manually by extending the wires from both ends by hand, while a ruler was used for references. Stretching a 2 cm long microwire to a length of 22 cm causes 1000% stretching. From Figure 5b, none of the as-extruded wires produced without thermal drawing endured more than 900% stretching, mainly limited by the mechanical failure where the SEBS shell broke. As opposed to that, the microwires produced with 300.1 mm s^−1^ drawing speed demonstrated electrical failure where the SEBS shell remained intact but the LM core experienced discontinuity. While eight (8) of these ten (10) microwires failed within a range of 700% to 950%, two (2) microwires sustained such extreme stretch. On the other hand, only one (1) of the ten (10) microwires produced with 70.1 mm s^−1^ drawing speed exhibited electrical failure at 950% stretching, while the rest of these microwires lasted to 1000% stretching.

### 3.4. Electrical Property with Wire Kinking

Because the conductive material remains in its liquid state, these LM microwires offer unprecedented flexibility and twistability while maintaining their mechanical and electrical properties. However, when these microwires were subjected to a sharp kink, their resistance is greatly affected. To observe this effect, a custom setup, shown in Figure 5c, was prepared using a surface mount hinge and a 3D-printed angular scale. After a microwire is attached to this custom setup using adhesive, as shown in the Figure, when the rotary plate of the hinge is closed, a kink of the desired angle can be formed on the wire. The angular scale is graded from 0° (full severed) to 90° (perpendicular bending) with 5° increments. When the rotary plate is fully closed with the fixed plate, a 0° kink is formed that actually breaks the LM core causing an open circuit. As soon as the kink angle is reduced, the LM core self-heals and produces a stable, continuous connection back, an extraordinary property of this smart material [23,24]. The change in electrical resistance of 6 cm long microwires when subjected to kinking and un-kinking processes having a single kink of different angles is depicted in Figure 5d. The as-extruded wires without thermal drawing showed an increase in resistance after a kink of 25° was produced, then the resistance jumped as kinks of higher angles were formed. On the other hand, the microwires produced with 300 mm s^−1^ drawing speed did not show a change in resistance until a kink of 15° was formed. All the microwires demonstrated an exponential rise in resistance at smaller kink angles, leading to severe conditions for 0° kinks. For all microwires, the coincidence of kinking and un-kinking resistance data suggests the mechanical robustness and stability of the microwires under such loading conditions [25,26].

### 3.5. Sewability of the Microwires

**Figure 6:**
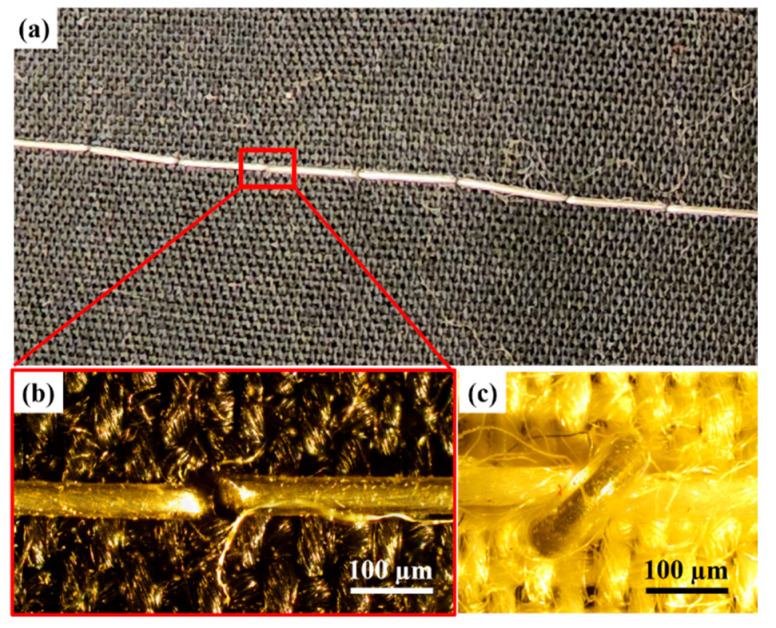
(**a**) Sewn microwire on traditional cotton fabric, as a proof of concept; (**b**) stitch knot from the bottom of the fabric; (**c**) stitch knot from the top side of the fabric.

These extremely stretchable and flexible microwires with metallic conductivity offer huge potential in the field of wearable electronics and smart sensing applications. Because of this, the ability to pattern these microwires on a substrate has a unique advantage [27,28]. To achieve this, these microwires were qualitatively tested using a manual sewing machine, with a straight type of stitch and a stitch length of 2–3 mm, to characterize the sewability of these microwires on fabrics or any other desired soft substrates. This qualitative test was targeted to understand the ability of these microwires to perform on a sewing machine without breaking and skipping stitches. Because of the stretchability of the microwires, the bobbin that is used for traditional threads was loosened to allow LM microwires to slip through the clearance. While the top thread of the sewing machine was a typical cotton thread, the LM microwire, as a bottom thread, was fed from the bobbin. As a proof of concept, some patterns of LM microwire were sewn on a piece of cotton fabric, as shown in Figure 6a. The microscopic images of the stitch knot of LM microwire from the bottom and top sides of the fabric are shown in Figure 6b and Figure 6c, respectively. The LM core is visible and shows no sign of damage.

In the realm of stretchable wires, a significant obstacle to conductivity lies in their kinking behavior when subjected to bending. As depicted in Figure 5d, kinks manifest at bend angles below 20°, causing interruptions in electrical flow. Conversely, bend angles exceeding 20° produce a curvature radius instead of kinks, preserving the wires’ conductivity. Consequently, during the sewability assessment, microwires were intentionally curved rather than kinked to maintain their electrical integrity, as the microwires were bent for angles less than 90° but far more than 20°.

## 4. Conclusions

To summarize, this article demonstrates a novel and scalable technique to produce LM microwires with an LM core diameter as small as around 52 µm and a SEBS elastomeric shell with a thickness of around 46 µm. The study also found that the SEBS extrusion speed, LM injection rate, and extrusion temperature play an important role in core–shell dimensions. The systematic analyses performed in this work suggest that the microwire sizes could be reduced by using a smaller size nozzle and needle system. While microwires with smaller core sizes exhibited higher electrical resistance, they were found to have similar resistivity with only slight variation. While all the microwires went through a minimum of 700% stretching without compromising mechanical or electrical properties, the microwires produced with 70.1 mm s^−1^ drawing speed demonstrated exceptional robustness by enduring 1000% stretching. The microwires were subjected to twisting and showed no noticeable effect on electrical performance. To test their flexibility, the microwires were subjected to a single loop kink of different angles which shows that the resistance of the microwires does not change until a kink of 15° angle is formed. With a kink of 0° angle, the microwires were severed and self-healed immediately after un-kinking. The microwires were tested under standard room conditions after several months. However, no significant aging effect or decay in electrical conductivity was observed. These experiments prove the suitability of these microwires in the field of stretchable and flexible electronics if they can be patterned effectively on a soft, stretchable substrate. To do that, as a proof of concept, these microwires were used in a manual sewing machine to test their sewability. Therefore, these microwires offer huge potential not only in wearable electronics applications, but also in the fashion industry and smart textiles in the form of high-performance conductive threads.

## Data Availability

The original contributions presented in the study are included in the article/Supplementary Material, further inquiries can be directed to the corresponding author.

## Supplementary Materials

The following supporting information can be downloaded at: https://www.mdpi.com/article/10.3390/ma17112770/s1. Figure S1: Engineering drawing file of the custom extruder nozzle. Figure S2: Engineering drawing file of the custom extruder nozzle (section view). Figure S3: Nozzle-Needle cross-section. Table S1: Drawing Speeds of the Microwires.

## References

[1] Liu Y., Pharr M., Salvatore G.A. (2017). Lab-on-Skin: A Review of Flexible and Stretchable Electronics for Wearable Health Monitoring. ACS Nano.

[2] Fernandez S.V., Cai F., Chen S., Suh E., Tiepelt J., McIntosh R., Marcus C., Acosta D., Mejorado D., Dagdeviren C. (2021). On-Body Piezoelectric Energy Harvesters through Innovative Designs and Conformable Structures. ACS Biomater. Sci. Eng..

[3] Beni G., Hackwood S., Jackel J.L. (1982). Continuous electrowetting effect. Appl. Phys. Lett..

[4] Whitney R.J. (1953). The measurement of volume changes in human limbs. J. Physiol..

[5] Khondoker M.A.H., Sameoto D. (2016). Fabrication methods and applications of microstructured gallium based liquid metal alloys. Smart Mater. Struct..

[6] Winter T.G. (2003). The evaporation of a drop of mercury. Am. J. Phys..

[7] Khondoker M.A.H., Ostashek A., Sameoto D. (2019). Direct 3D Printing of Stretchable Circuits via Liquid Metal Co-Extrusion Within Thermoplastic Filaments. Adv. Eng. Mater..

[8] Li G., Wu X., Lee D.-W. (2016). A galinstan-based inkjet printing system for highly stretchable electronics with self-healing capability. Lab Chip.

[9] Wang H., Li R., Cao Y., Chen S., Yuan B., Zhu X., Cheng J., Duan M., Liu J. (2022). Liquid Metal Fibers. Adv. Fiber Mater..

[10] Sangeeth C.S.S., Wan A., Nijhuis C.A. (2014). Equivalent Circuits of a Self-Assembled Monolayer-Based Tunnel Junction Determined by Impedance Spectroscopy. J. Am. Chem. Soc..

[11] Ladd C., So J.-H., Muth J., Dickey M.D. (2013). 3D Printing of Free Standing Liquid Metal Microstructures. Adv. Mater..

[12] Neumann T.v., Dickey M.D. (2020). Liquid Metal Direct Write and 3D Printing: A Review. Adv. Mater. Technol..

[13] Zhu S., So J.-H., Mays R., Desai S., Barnes W.R., Pourdeyhimi B., Dickey M.D. (2013). Ultrastretchable Fibers with Metallic Conductivity Using a Liquid Metal Alloy Core. Adv. Funct. Mater..

[14] Wu Y., Zhen R., Liu H., Liu S., Deng Z., Wang P., Chen S., Liu L. (2017). Liquid metal fiber composed of a tubular channel as a high-performance strain sensor. J. Mater. Chem. C Mater..

[15] Khondoker M.A.H., Sameoto D. (2019). Direct coupling of fixed screw extruders using flexible heated hoses for FDM printing of extremely soft thermoplastic elastomers. Prog. Addit. Manuf..

[16] He Y., Zhou L., Zhan J., Gao Q., Fu J., Xie C., Zhao H., Liu Y. (2018). Three-Dimensional Coprinting of Liquid Metals for Directly Fabricating Stretchable Electronics. 3D Print. Addit. Manuf..

[17] Badhe Y., Rocha-Flores P.E., Voit W.E., Remer D., Costella L., Joshi-Imre A. (2021). Lithographically patterned stretchable metallic microwiring on electrospun nanofiber mats. J. Vac. Sci. Technol. B.

[18] Chen S., Lou Z., Chen D., Shen G. (2018). Printable Zn_2_GeO_4_ Microwires Based Flexible Photodetectors with Tunable Photoresponses. Adv. Mater. Technol..

[19] Samokhin A. Open-Source Syringe Pump. Mass Spectrometry Research Group, Chemistry Department of Moscow State University 2020. https://www.mass-spec.ru/projects/diy/syringe_pump/eng/.

[20] Filastruder FILAWINDER 2018. https://www.filastruder.com/products/filawinder.

[21] Khondoker M.A.H., Sameoto D. Design and characterization of a bi-material co-extruder for Fused Deposition Modeling. Proceedings of the ASME International Mechanical Engineering Congress and Exposition, Proceedings (IMECE).

[22] Zhao Z., Soni S., Lee T., Nijhuis C.A., Xiang D. (2023). Smart Eutectic Gallium–Indium: From Properties to Applications. Adv. Mater..

[23] Palleau E., Reece S., Desai S.C., Smith M.E., Dickey M.D. (2013). Self-Healing Stretchable Wires for Reconfigurable Circuit Wiring and 3D Microfluidics. Adv. Mater..

[24] Morita L., Jalali S., Vaheb A., Elsersawy R., Golwala K., Asad A., Dolez P.I., Hogan J.D., Khondoker M.A.H., Sameoto D. (2023). Towards high efficiency and rapid production of room-temperature liquid metal wires compatible with electronic prototyping connectors. Micromachines.

[25] Jiao C., Wang Q., Li L., Chen W., Liu J., Xu Y., Song L., Fu S., Hu L. (2023). In situ 3D printing of Liquid Metal-hydrogel hybrid for Multifunctional Soft Bioelectronics and devices. Cell Rep. Phys. Sci..

[26] Hassan I., Selvaganapathy P.R. (2022). A microfluidic printhead with integrated hybrid mixing by Sequential Injection for multimaterial 3D printing. Addit. Manuf..

[27] Hur O., Tutika R., Klemba N., Markvicka E.J., Bartlett M.D. (2024). Designing liquid metal microstructures through directed material extrusion additive manufacturing. Addit. Manuf..

[28] Xing R., Huang R., Qi W., Kong J., Dickey M.D. (2024). Protocol for 3D and 4d printing of highly conductive metallic composite using liquid metal gels. STAR Protoc..

