# Supplementary material for: Scalable Microwires through Thermal Drawing of Co-Extruded Liquid Metal and Thermoplastic Elastomer
Source: Materials (Basel). 2024 Jun 6;17(11):2770. doi: 10.3390/ma17112770 (PMC11173905; doi:10.3390/ma17112770)
Supplement: Supplementary file 1 [file materials-17-02770-s001.zip › materials-3016908-supplementary.pdf]

Supplementary Information

Scalable Microwires through Thermal Drawing of Co-extruded Liquid Metal and Thermoplastic Elastomer

Section S1: The Detailed Drawing of the Custom Extruder Nozzle

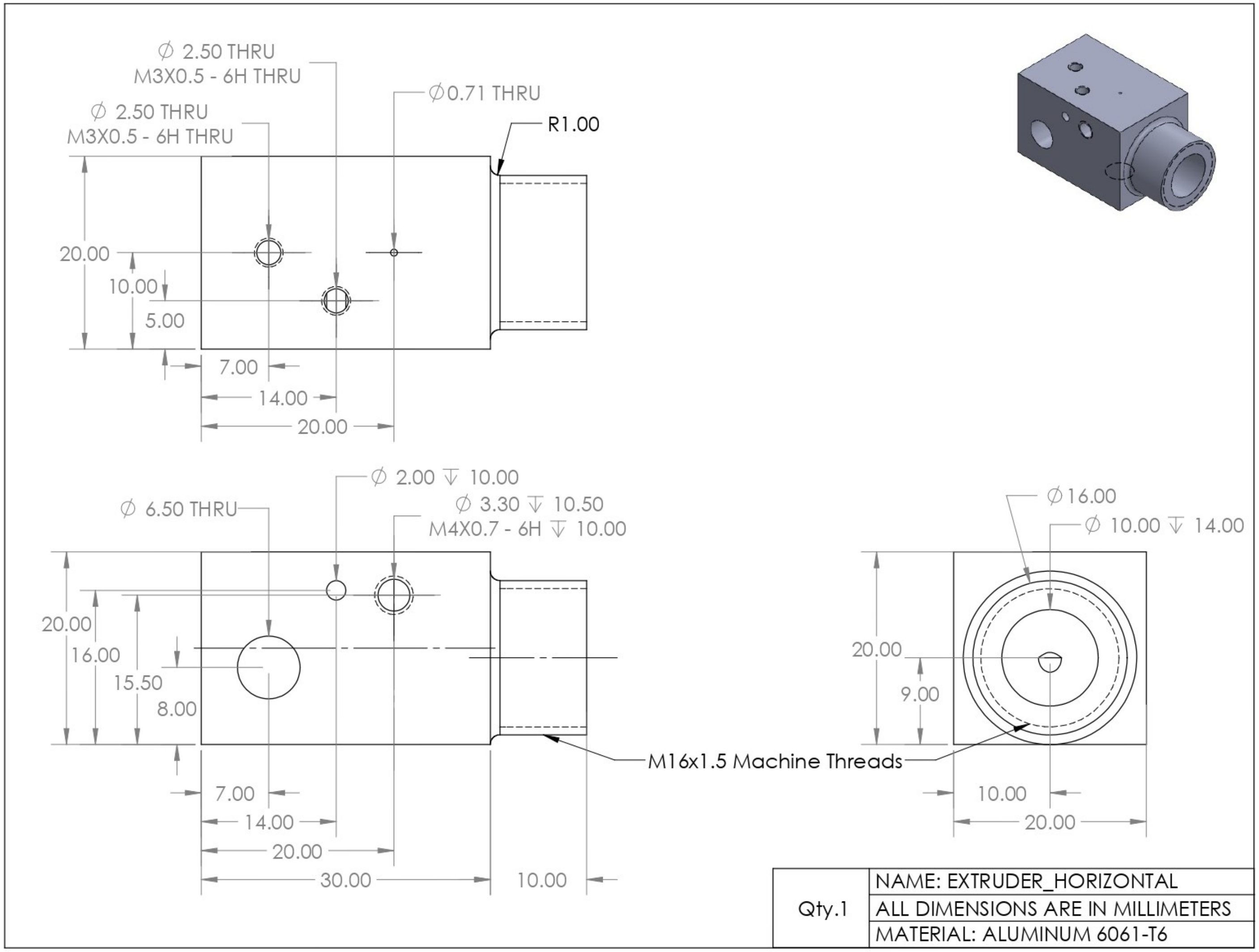

Figure S1: Engineering drawing file of the custom extruder nozzle

Supplementary Information

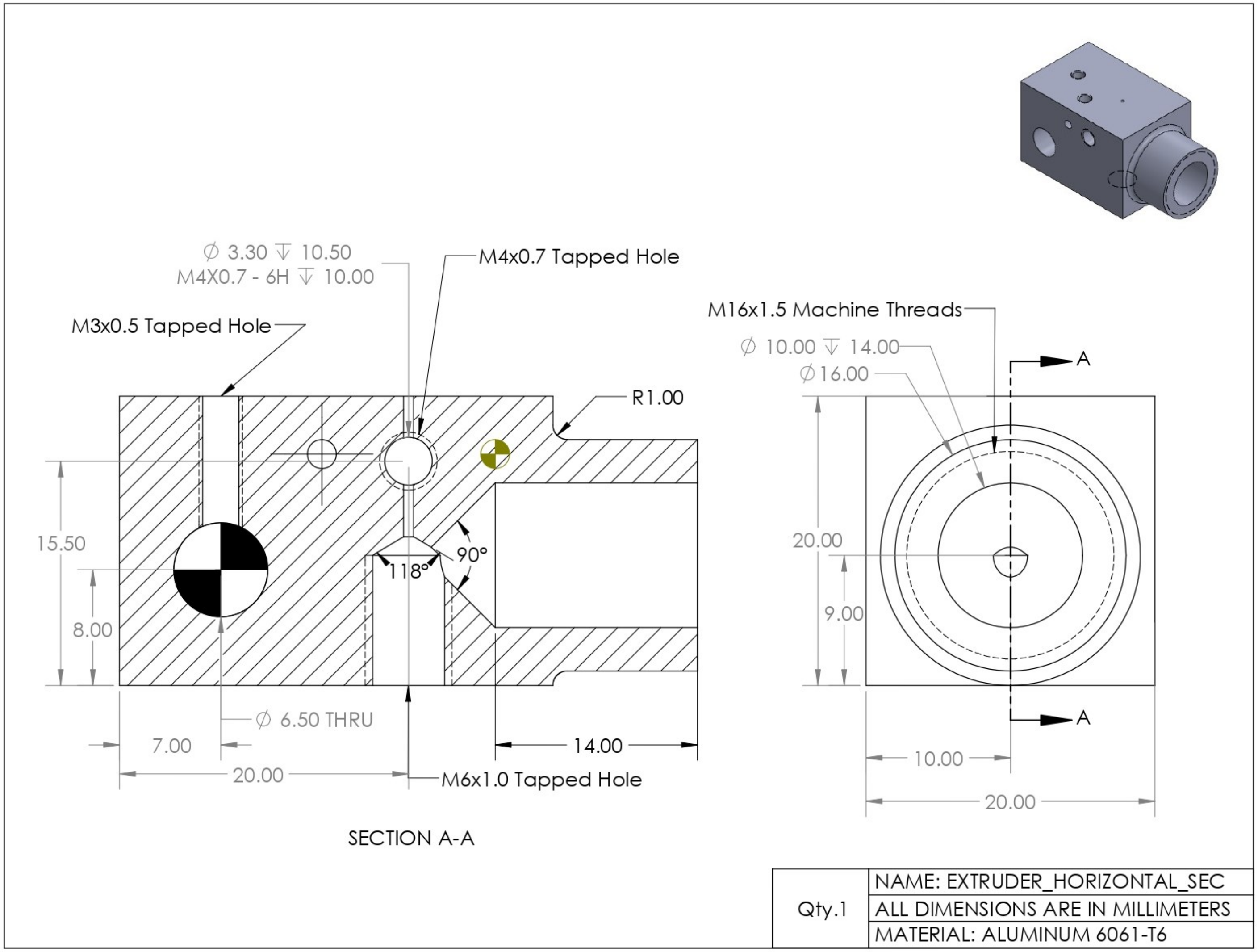

Figure S2: Engineering drawing file of the custom extruder nozzle (section view)

Supplementary Information

Section S2: The Calculation of Drawing Speeds from rpm

The three levels of the rpm of the winding system were tested, and they are 0 rpm, 15 rpm, and 67 rpm. Table S1 will explain how the drawing speeds of the microwires are calculated from the rpm of the winding system.

The spool diameter = 85 mm

Table S1: Drawing Speeds of the Microwires

	Filawinder Speed (A)	Angular Speed $\left(B = \frac{2\pi A}{60}\right)$	Spool Diameter (C)	Drawing Speed $\left(D = B \times \frac{C}{2}\right)$
1.	0 rpm	0 rad/s	85 mm	0 mm/s
2.	15.73 rpm	1.65 rad/s	85 mm	70.1 mm/s
3.	67.42 rpm	7.06 rad/s	85 mm	300.1 mm/s

Section S3: SEBS Extrusion Speed and LM Injection Speed

Nozzle ID: 1 mm

Needle OD: 0.7112 mm

Needle ID: 0.4064 mm

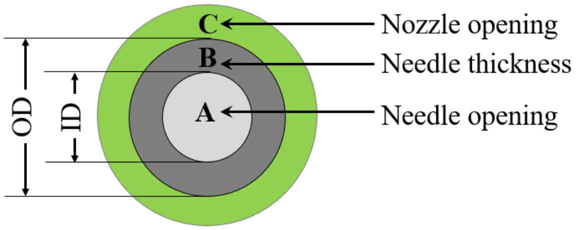

Figure S3: Nozzle-Needle cross-section

Area A will be occupied by LM, area C will be fill-up by SEBS, and area B is the gap between these two areas due to the thickness of the needle wall. In this experiment, the SEBS extrusion speed and LM injection speed will be adjusted in such a way that, the ratio of these speeds equals the cross-sectional area of their flow, i.e. the ratio of Area C and Area A.

Supplementary Information

Therefore,

Area A = $\pi\left(\frac{0.4064}{2}\right)^2$ = 0.13 mm^2

Area C = $\pi\left(\frac{1}{2}\right)^2-\pi\left(\frac{0.7112}{2}\right)^2$ = 0.39 mm^2

The ratio of Area C: Area A = 0.39: 0.13 = 3: 1 . Therefore, the three pairs of tested speeds are:

	SEBS Extrusion Speed	LM Injection Speed
1.	1.50 mm^3/s	5×10^5 μL/s
2.	3.00 mm^3/s	10×10^5 μL/s
3.	4.75 mm^3/s	15×10^5 μL/s
